# A newly developed tool for measuring the availability of human resources for emergency obstetric and newborn care services: prospective analytic study in two district-level public facilities in Bangladesh

**DOI:** 10.1186/s12913-018-3511-1

**Published:** 2018-09-04

**Authors:** Taposh Kumar Biswas, Anjuman Ara Begum, Shamima Akther, M. Hafizur Rahman, Henry B. Perry, Heidi E. Jones, Mahbub Elahi Chowdhury

**Affiliations:** 1 Health Systems and Population Studies Division, International Centre for Diarrhoeal Disease Research, Bangladesh (icddr,b), 68 Shaheed Tajuddin Ahmed Sarani, Mohakhali, Dhaka, 1212 Bangladesh; 2 Johns Hopkins University Bloomberg School of Public Health, Baltimore, MD USA; 3 CUNY Graduate School of Public Health & Health Policy, New York, USA

**Keywords:** Bangladesh, Human resources, Index, Maternal and newborn care, Round-the-clock services, District hospital, Mother and child welfare Centre

## Abstract

**Background:**

In Bangladesh, while the infrastructure of public health facilities to provide maternal and newborn care services is adequate, services are not always available due to insufficient staffing. A human resource availability index for health facilities is needed for monitoring and advocacy. This study aimed to develop indices for measuring the availability of different types of human resources to provide round-the-clock emergency obstetric and newborn care (EmONC) service at district-level public facilities.

**Methods:**

As part of a larger intervention study, 30 days of prospective observation of providers was done at a district hospital (DH) and a mother and child welfare centre (MCWC) in one district of Bangladesh using checklists. A scoring system was developed to create an index to quantify the availability of providers for maternal and newborn care.

**Results:**

Based on the newly developed index, medical doctors in the emergency department of the DH were 100% available, but ranged from 27 to 41% availability in the obstetrics/gynecology (ob/gyn) and pediatric wards. In MCWC, the corresponding indices ranged from 32 to 36%. In the DH, the availability of nurses in the ob/gyn ward (96%) was relatively better than in the pediatric ward (65%) but that in operation theatre was only 31%. In the MCWC, the index for the presence of a paramedic or nursing aid was 82% in the ob/gyn ward and 63% in the operation theatre. However, the availability scores of facility support staff for maintenance and security were generally high (over 90%) in both facilities.

**Conclusions:**

Our newly developed index on availability of providers demonstrated huge gaps in availability of providers in evening and night shifts in most of the disciplines in the study facilities. This provider availability index is easy to create and can be used as a meaningful tool to quantify gaps in human resources by type in various types of district-level health facilities. Further studies are needed for adaptation of this tool in different types of health facilities and to assess its implication as an advocacy tool.

## Background

Bangladesh has made substantial progress in reduction of maternal mortality from 569 per 100,000 live births in 1990 to an estimated 176 per 100,000 live births in 2015 [1]. Partly, this reduction is due to a substantial increase in facility-based deliveries that enables effective management of obstetric complications [2]. Though this achievement has been obtained despite many weaknesses in the Bangladesh health system, further system strengthening is warranted to achieve the sustainable development goal (SDG) target to reduce the maternal mortality ratio below 80 per 100,000 live births by 2030 [3].

The country has a sufficient number of health facilities to provide emergency obstetric and newborn care (EmONC) services [4]. In the public sector, in each district, there is a district hospital (DH) and a mother and child welfare centre (MCWC) to treat obstetric and newborn health complications and to offer delivery and routine maternal and newborn health (MNH) care services. There are also an adequate number of EmONC facilities at the sub-district levels (accessible within one hour travel time) and MNH care facilities at lower levels [5]. However, despite having the infrastructure, a major proportion of these public facilities are struggling to provide the required services due to lack of human resources [5]. About one-third of public facilities were found not to provide full range of EmONC services in 2012 mainly due to the unavailability of obstetric and anesthesia specialists to provide surgical intervention [5]. Also there is a major shortage of nurses and paramedics to provide basic emergency obstetric and other MNH care services [6].

A number of health system strengthening initiatives including the “MaMoni” project of Save the Children have been introduced in an effort to reduce the human resource gap in public facilities [7]. The “MaMoni” project is being implemented for an integrated safe motherhood, newborn care and family planning services to strengthen the health system in Habiganj districts in Bangladesh starting in June 2010 [7]. Under this project temporary contractual staff have been provided to assist with necessary stocking of drugs, management of emergency blood transfusion, and arrangement of emergency transport for referrals [7]. As part of this project, 10 nurses were supported by the project in the DH and one nurse in the MCWC in Habiganj district to strengthen obstetrics/gynecology (ob/gyn) and pediatric care services.

The utilization of services depends on the availability of providers as well as the accessibility, acceptability and quality of health services and healthcare providers [8]. However, to our knowledge, no study has systematically quantified the availability of providers for round-the-clock EmONC and other MNH care services in public facilities in Bangladesh. While routine MNH care services are generally provided during the morning shift, emergency services should be available 24 h a day. Furthermore, an effective service delivery system should have the minimum required number of skilled providers available at the health center and remain on-call for emergencies. Maintaining appropriate staffing is difficult due to the complex nature of the minimum required number of staff with different skills across shifts and the challenge of monitoring the performance of a health facility in terms of appropriate staffing.

An index that quantifies the availability of providers in health facilities is needed for managers and policy makers. Such an index may also be useful as an advocacy tool to promote policies to reduce human resource gaps in health facilities. The current study developed a human resource availability index for staff needed to ensure 24 h EmONC, accounting for multiple types of providers at district level public health facilities in Bangladesh.

## Methods

As part of a larger intervention study, prospective observational data was collected at the DH and MCWC of one district in Bangladesh, from February 2014 to May 2015. The study protocol was approved by the Institutional Review Board of icddr,b.

### Data collection process

Observations on the availability of service providers were carried out over a 30-day period divided in three phases. Each phase consisted of 10 consecutive days of observation including weekends at each study facility. According to regulations, the providers report to work for three shifts: morning (8 AM-2 PM), evening (2 PM–9 PM) and night (9 PM-8 AM). Information on the availability of different types of providers of maternal and newborn care services in various departments of the study facilities (covering ob/gyn, pediatrics, and emergency department; laboratory and pathology services) was collected through observation checklists. At each study facility, one team consisting of a trained medical doctor and paramedic, hired from outside of the study facility was engaged in data collection. The study medical doctor collected data by observing the presence of medical doctors (consultants, medical officers, anesthetist) at the health facilities and the paramedic was responsible for observing the availability of nurses and other technical and support staff such as family welfare visitors (FWVs, a type of paramedic), medical technologists, assistant nursing attendants (ANAs), nurse aides (*Dai nurses*), ward boys, cleaners (*aya*), sweepers, errand people (peons) and guards. Observations on the presence of the above service providers were collected at each of the three shifts (morning, evening and night). The details of the type of data collected by observing various providers are shown in Table 1.

**Table 1:** Type of availability data collected for various providers

Type of provider	Type of availability data collected
Medical doctor	a) Physically present more than 50% of the time during the assigned shift b) Physically present less than 50% of the period during the assigned shift c) Remained off-site, but responded appropriately to an emergency call (i.e. came to the facility to manage the patient) during the assigned shift d) Remained off-site, and did not respond appropriately to an emergency call (i.e. did not come to the facility to manage the patient) during the assigned shift
Consultant (ob/gyn)	
Consultant (pediatrics)	
Anesthetist	
Resident medical officer	
Medical officer (indoor)	
Medical technologist	
Other Technical staff	a) Presence of the number of each staff category in different duty shift
Nurse	
Family welfare visitor (FWV)	
Assistant nursing attendants (ANA)	
Nurse aid	
Support staff	a) Presence of the number of each staff category in different duty shift
Ward boy	
Sweeper	
Cleaner (aya)	
Errand people (peon)	

In some cases, to collect the data on presence of the providers in the night shift, the relevant information was collected by interviewing the service providers who worked in the respective department during the previous night. For medical doctors, they were coded as having responded appropriately to an emergency call if they came to the facility in response to the call, and not appropriately if they did not come to the facility to manage the case. At district-level health facilities in Bangladesh, medical doctors who are not assigned to the emergency department are supposed to be present for the morning shift during work days and to cover the remaining hours (evening and night shifts and whole holidays) off-site until they are called-in as needed.

### Operational definition of variables

To provide emergency obstetric care services adequately, the minimum required number of staff for each shift, was defined through consultation with the service providers of the respective study facilities. To assess the caseload, the service data from the three months preceding the observation were reviewed to assess number of deliveries, number of maternal and newborn complications management, number of clients receiving family planning during the three different shifts of the day to ensure that caseload was taken into account when defining the needed staff. From DH, on average 3–4 delivery care, 2–3 obstetric complication management and 3–4 sick newborn care services were provided in different shifts of a given day. From MCWC, on average, about 1 delivery care service was provided per shift in a day. In addition, in the morning shift, from DH, 10 routine maternity care (antenatal and postnatal) services and from MCWC, 5 routine maternity care and 10 family planning services were provided.

For calculation of availability index of medical doctors in DH, consultants and medical officers were combined as having the minimum required competency in ob/gyn. Presence for medical doctors and medical technologists was defined as being present at the facility for more than 50% of the duration of the assigned shift for on-site shifts and coming to the facility after responding to an emergency call for off-site shifts. The minimum required number of nurses/FWVs was defined as at least three nurses in the morning and two in each of the evening and night shifts for the ob/gyn and pediatric wards. For the operating theatre (OT), the minimum requirement of nurses was defined as presence of at least two nurses in each of the three shifts. For support staff (peon/aya/sweeper/guard), the presence of at least two (of any combination of providers in these categories) for each shift was considered to be necessary to provide full services.

### Data analysis

To create an index of the availability of different types of providers in the health facilities, we analysed the observational data in two steps. In the first step, we estimated the percentage of availability of different types of providers across three different shifts. For medical doctors and technologists, these percentages were calculated based on the presence of individual providers from their respective discipline. Availability of nurses and other support staff were assessed by the presence of the predefined minimum required number of providers in different shifts. For estimating the availability of nurses, we combined government nurses with the additional nurses provided by the MaMoni project. In the second step, we analysed the availability of care providers in the study facilities after compensating for deficiencies from the set standards for different categories of providers. Thus, the availability index for medical doctors was defined as- (a + b/2 + c - d)/N *100.

where:

a = number of shifts for which at least one medical doctor was present for more than 50% duration of the shift.

b = number of shifts for which at least one medical doctor was present for less than 50% duration of the shift.

c = number of shifts for which at least one medical doctor presented to the facility in response to any emergency call.

d = number of shifts for which no medical doctor presented to the facility in response to any emergency call.

N = total number of shifts.

For the above analysis, we assumed that at least one medical doctor (either consultant or medical officer) was required in the relevant disciplines to offer obstetric and newborn care. This decision was based on the governmental organogram for district level health facilities in Bangladesh that include one post of consultant for ob/gyn, pediatrics and anesthesia respectively.

For nurses/FWVs, if a smaller number was present than that considered for full presence in a shift, availability was calculated by applying the proportion of staff present. Thus, if two nurses were present in the morning shift (where three were needed), the shift was considered to have two-thirds presence. Thus, the availability indices for nurses/FWVs for both the ob/gyn and pediatric ward were defined as follows:

For the morning shift = (m_3_ + 2 m_2_/3 + m_1_/3- m_0_)/N*100.

For the evening and night shifts = (m_2_+ m_1_/2-m_0_)/N*100.

For OT nurses for all shifts the availability index = (m_2_+ m_1_/2+ m_2a_ + m_1a_/2- m_0_)/N*100.

where:

m3 = number of shifts in which at least three nurses were present.

m_2_ = number of shifts in which at least two nurses were present.

m_1_ = number of shifts in which at least one nurse was present.

m_0_ = number of shifts in which no nurse was present.

m_2a_ = number of shifts in which at least two OT nurses were present in response to any emergency call.

m_1a_ = number of shifts in which at least one OT nurse was present in response to any emergency call.

N = total number of shifts.

Availability indexes for medical technologists and ANAs used the same algorithm as that for medical doctors.

The availability index for support staff (peon/aya/sweeper/guard) was defined as (s_2_+ s_1_/2-s_0_)/N*100.

Where:

s_2_ = number of shifts in which at least two support staff were present.

s_1_ = number of shifts in which at least one support staff was present.

s_0_ = number of shifts in which no support staff was present.

## Results

This study documented a substantial shortage of providers in both the DH and the MCWC for providing EmONC and other MNH care services. As shown in Table 2, the numbers of posts (or positions available) for providers are not adequate to provide round-the-clock services. For instance, at least three medical doctors are needed to ensure presence of at least one in each of morning, evening and night shifts. Further, for some types of providers, those hired (in-position) was less than the allocated posts showing unmet recruitment targets. In both DH and MCWC, only one specialist medical doctor was allocated for ob/gyn services. In the DH, although general medical officers are available, no such position exists at the MCWC. Only one provider (MO Clinic) is responsible for round-the-clock services in this facility. In the DH, only seven of 14 allocated medical officers were available to provide outpatient services. For pediatrics, though two consultants were present to provide services for both inpatients and outpatients, no medical officer was assigned to work with them. There were also two resident medical officers who assisted in providing emergency services along with one emergency medical officer and other medical officers. In the DH, there were also substantial shortages of the other technical and support staff (senior staff nurses, ayas, guards and sweepers). However, in the MCWC, measures had been taken to reduce these shortages by bringing additional providers from other facilities.

**Table 2:** Distribution of number of posts and in-positioned providers for maternal and newborn care in the study facilities

Type of providers	Number of post/in-position by provider and facility type			
	DH		MCWC	
	Post	In-position^a^	Post	In-position^a^
Medical doctor				
Consultant (ob/gyn)	1	1		
Medical officer (trained)	–	1	1	1
Consultant (anesthesia)	1	0	–	
Anesthetist/medical officer (trained)	1	1	1	1
Consultant (pediatrics)	1	2	–	
Resident medical officer	2	2	–	
Pathologist	1	1	–	
Emergency medical officer	3	1	–	
Medical officer for outpatients	14	7	–	
Other technical staff				
Senior staff nurse	38	31	–	
Staff nurse (provided by the MaMoni Project)	–	10	–	1
Medical technologist (laboratory)	1	2	–	
Family Welfare Visitor	–	–	1	6
Assistant nursing attendant/dai nurse	–	–	2	3
Other support staff				
Peon/ward boy	17	18	1	2
Guard	4	1	–	2
Aya/maid	10	7	–	1
Sweeper	19	13	1	3

DH = district hospital, MCWC = mother and child welfare centre, ob/gyn = obstetrics and gynecology

^a^Higher numbers for some in-positioned providers than the number of posts are due to bringing in additional providers of that category from other facilities

**Fig. 1:**
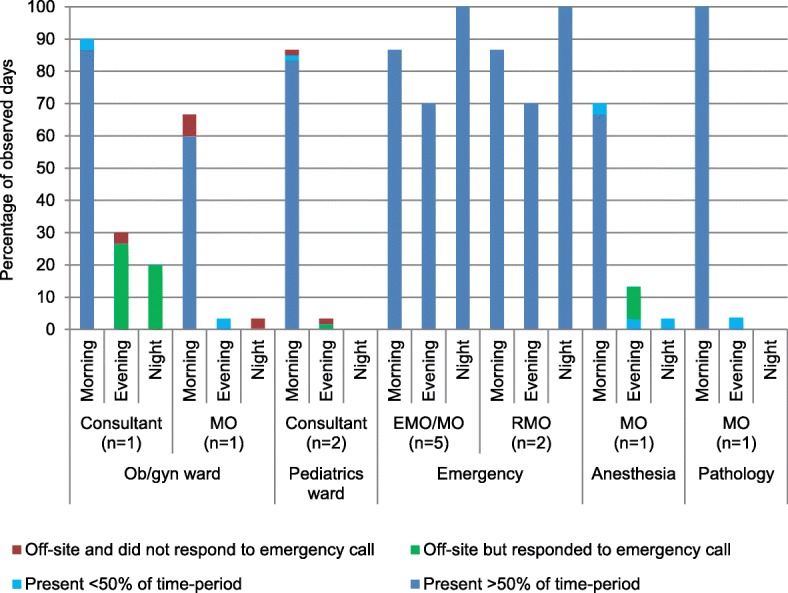
Pattern of availability of medical doctors in different shifts of a day in DH. Legend: DH = district hospital, MO = medical officer, RMO = resident medical officer, EMO = emergency medical officer, ob/gyn = obstetrics and gynecology

As shown in Fig. 1, in the DH, the doctors were mostly available in the morning shifts and their availability was very poor during evening and night shifts to respond to emergency calls. In the ob/gyn ward of the DH, the consultant was present in 87% of the morning shifts but during the evening and night shifts, their availability for emergency call was less than 30%. The medical officer (ob/gyn) was present only in 60% of the morning shifts and rarely responded to emergency calls in the evening and night shifts. Similarly, for the pediatric ward, although the consultant was mostly present (83%) in the morning shifts, response to emergency calls during the evening and night shifts was almost non-existent. The medical officer (trained in anesthesia) was present during 70% of the morning shifts and responded to only a small percentage of emergency calls in the evening and night shifts. The pathologist was present during all the morning shifts throughout the 30-day observation period but failed to respond to emergency requests during other shifts. The physicians in the emergency department, however, generally provided round-the-clock services as required.

As shown in Table 3, the presence of nursing and support staff was strong during the daytime for the ob/gyn and pediatric wards and the operating theatre, but major staffing gaps were apparent in the evening and night shifts. In general, availability of nurses in the pediatric ward was the weakest. Staffing of other support staff was consistently strong round-the-clock. In the ob/gyn ward of the DH, in 80–97% of the shifts the minimum expected number of nurses was present according to the operational definition of this study. But, in the pediatric ward and operation theatre, the availability of nurses was much less than the expected minimum number, particularly in the evening and night shifts. Similarly, the availability of a medical technologist was also poor in the evening and night shifts. However, the availability of other support staff, (such as peon, sweeper and aya) was considerably better, as at least two (from any of the above categories) were present in 100% of morning and evening shifts and 80% of the night shifts.

**Table 3:** Availability of nurses, other technical and support staff in DHby shift of the day

Number of providers by type and department	Percentage of days present by shift (*n* = 30)		
	Morning	Evening	Night
Obstetric/gynecology ward			
> =3 nurses present	96.7	26.7	6.7
2 nurses present	3.3	70.0	73.3
1 nurse present	0.0	3.3	20.0
Pediatric ward			
> =3 nurses present	13.3	0.0	0.0
2 nurses present	66.7	23.3	40.0
1 nurse present	20.0	76.7	60.0
Operating theatre			
> =2 nurses present	83.3	0.0	0.0
1 nurse present	6.7	0.0	0.0
> =1 nurse attended on emergency call	0.0	13.3	0.0
Medical technologist (laboratory)			
Present	90.0	0.0	0.0
Responded when called for an emergency	6.7	83.3	73.3
Support staff			
> =2 peons/MLSSs/ayas/sweepers present	100.0	100.0	80.0
1 peon/MLSS/aya/sweeper present	0.0	0.0	20.0

MLSS = Member of lower subordinate staff

**Fig. 2:**
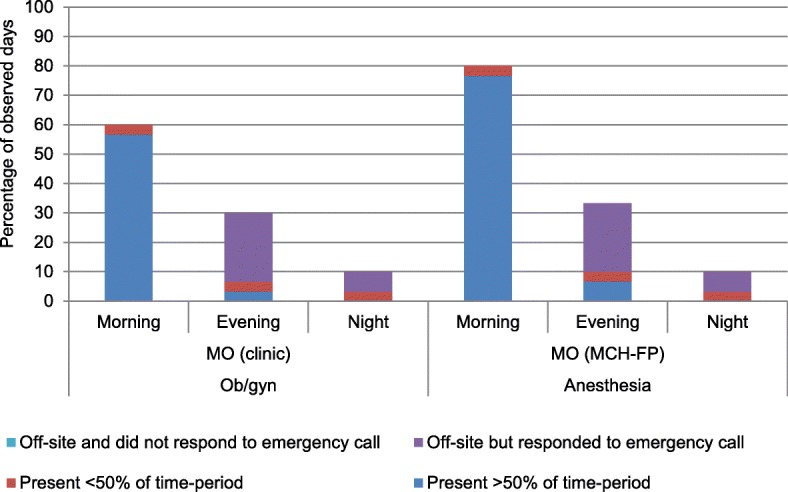
Pattern of availability of medical doctors in different shifts of a day in MCWC. Legend: MCWC = mother and child welfare centre, MCH-FP = maternal and child health-family planning, ob/gyn = obstetrics and gynecology

As shown in Fig. 2, in MCWC, the availability of medical doctors was poor overall and the situation was even poorer in evening and night shifts. The MO (clinic) was present in about 60% of the morning shifts and responded to emergency calls in about 23% of the evening and 7% of the night shifts. The presence of MO (MCH-FP) for providing anesthesia services was slightly higher in the morning shift compared to that of the MO (clinic). However, in the evening and night; their availability patterns were similar and much lower than the morning shift.

As shown in Table 4, in the MCWC, other than the morning shift, the availability of FWVs and other technical and support staff was also poor. Though at least 3 FWVs were present for 90% of the morning shifts, during the evening and night shifts at least 2 FWVs were present in 67% and 40% of the shifts respectively. However, availability of nurse aides (ANA/dai nurse and female medical attendants) compensated for some of the shortages of FWVs during the evening and night shifts.

**Table 4:** Availability of FWVs as well as other technical and support staff in MCWC by shift of the day

Type of providers	Percentage of days present by shift (*n* = 30)		
	Morning	Evening	Night
Family welfare visitor (FWV)			
3 FWVs present	90.0	16.7	3.3
2 FWVs present	6.7	50.0	36.7
1 FWV present	3.3	33.3	56.7
No FWV present	0.0	0.0	3.3
Other technical staff			
ANA/dai nurse	90.0	43.3	56.7
Female medical attendant	100.0	–	–
Pharmacist	90.0	–	–
Support staff			
At least 1 peon/guard and 1 sweeper/aya	100.0	73.3	90.0
1 peon/guard/sweeper/aya	0.0	26.7	10.0

FWV=Family welfare visitor, ANA = Assistant nursing attendant

The index on availability of providers demonstrates major gaps in the presence of providers in the evening and night shifts for most of the staff categories (Table 5). In the DH, in the morning shift availability index of the medical doctors in all the disciplines except anesthesia was above 83%. However, in the evening and night shifts, except for the emergency department, in all other disciplines, the availability index of medical doctors was below 20%. In MCWC, the availability index of doctors in the morning shift was even lower (58.3% to 68.3%).

**Table 5:** Index on availability of providers by shift of a day in the study facilities

Type of providers	Index (%) on availability of providers by facility by shift of a day			
	Morning	Evening	Night	Day average
District hospital				
Medical doctors				
Obstetrics/gynecology ward	83.3	21.7	16.7	40.6
Pediatric ward	86.7	0.0	0.0	28.9
Emergency department	100.0	100.0	100.0	100.0
Anesthesia department	68.3	11.7	1.7	27.2
Pathology department	90.0	0.0	0.0	30.0
Nurses				
Obstetrics/gynecology ward	98.9	98.3	90.0	95.8
Pediatric ward	64.4	61.7	70.0	65.4
Operation theatre	86.7	6.7	0.0	31.1
Other technical and support staff				
Medical Technologist	96.7	83.3	73.3	84.4
Ward boy/MLSS/Aya/Peon	100.0	100.0	90.0	96.7
Mother and child welfare centre (MCWC)				
Medical doctors				
Obstetrics/gynecology ward	58.3	28.3	8.3	31.7
Anesthesia department	68.3	31.7	8.3	36.1
FWVs/ANA/Dai Nurse				
Obstetrics/gynecology ward	96.7	95.6	83.3	82.4
Operation theatre	90.0	43.3	56.7	63.3
Other support staff				
Ward boy/MLSS/Aya/Peon	100.0	86.7	95.0	93.9

FWV=Family welfare visitor, ANA = Assistant nursing attendant, MLSS = Member of lower subordinate staff

In the DH, there was a major shortfall of nurses for the operation theatre, though the staffing situation of nurses was relatively better in the MCWC. In both the DH and the MCWC, the availability index for the other technical and support staff was comparatively better than in the higher staffing categories (Table 5).

## Discussion

The availability of EmONC services depends on ensuring a sufficient number of skilled human resources at each public facility to provide adequate care for the caseload. However, in most developing country contexts, it is quite challenging to ensure availability of the required human resources in the health facilities. Inadequate number of posts, high number of vacant posts [6, 9] and frequent absenteeism [10] are major challenges to ensuring emergency services. Further, a weak monitoring and supervision system fails to identify deficiencies in human resources to inform higher-level program managers and policy makers on corrective actions. Our newly developed tool identified gaps in availability of minimum required staffing for EmONC services in two district-level public facilities in Bangladesh. This new tool can also serve as an advocacy instrument to sensitize policy makers about the need to strengthen governance and accountability for effective management of human resource issues for EmONC services in public facilities in Bangladesh.

Consistent with other study findings [6, 9], our study documented a substantial number of vacant posts in district-level public facilities to provide EmONC services. Furthermore, as we have shown elsewhere, the currently allocated number of staff was not sufficient to meet service demand [5, 6]. Moreover, our study highlighted the largest shortages for staff availability during the evening and night shifts, leading to insufficient service availability for many emergency cases. As a result of understaffing, providers on duty during the morning shift might be less inclined to respond to emergency calls during the evening and night shifts. In each DH and MCWC, at least two additional providers are needed to ensure 24 h coverage of services.

For anesthesia, there were two positions at the DH: one for a specialist (consultant) and the other for a trained medical officer. However, during the study period, the post of consultant-anesthesia was vacant. At the MCWC, the provider who was assigned for the same service was overburdened with additional responsibilities such as providing surgical family planning methods (sterilization) and supervising family planning activities. Due to the unavailability of a post for anesthesia consultant at MCWC, the trained medical officer was the only person providing anesthesia services. Thus, only in about one-fourth of a typical 24-h day at the DH and one-third of a typical 24-h day at the MCWC were anesthesia services available. To ensure full coverage, in addition to filling the vacant posts, several additional posts need to be created and filled urgently.

The lack of availability of pediatric service providers was due to either an inadequate number of providers or gaps in coordination among the existing providers. In the MCWC, there was no dedicated provider for pediatric services. In the DH, though a special arrangement was made to assign an additional pediatric consultant, no medical officer was assigned to support the existing specialists for inpatient services. Among the two pediatric consultants at the DH, one was engaged for inpatient services and the other for outdoor services. Thus round-the-clock availability of providers for pediatric services was at stake. Improved coordination among the existing providers is warranted to ensure round-the-clock pediatric services in the DH. For the MCWC, a new post of medical doctor for pediatric care is recommended.

Despite the support of the MaMoni project providing additional nurses in the ob/gyn and pediatric wards, both the DH and MCWC had additional requirements to ensure round- the-clock service availability. In the case of OT, since there was less availability of the medical doctors during the evening and night shifts, the nurses’ availability was also lower as both of those groups remained off-site even though they were supposed to be present after responding to any emergency call during those shifts. To ensure the availability of surgical services round-the-clock, medical doctors, nurses and other support staff need to be present at all times.

Our index of human resource availability clearly demonstrates that the current human resource configuration is insufficient to ensure adequate round-the-clock EmONC services in the district-level public facilities. The existing staffing structures do not reflect the requirements of actual service demand and priorities. Our tool has merit to quantify gaps in availability of providers during the different shifts.

Our findings are supported by those of the 2015 State of the World’s Midwifery report which identified Bangladesh as having a critical shortage of doctors, nurses and midwives [11]. Moreover, our doctor:nurse ratio of 2:1 [12] is also the reverse of the WHO recommended ratio of 1:3 [13]. To minimize these shortages, the government has been recruiting a greater number of medical doctors every year, especially those who are to be posted at the district-level facilities where the need is the greatest. Other initiatives are also needed urgently to recruit additional nurses and creation of positions for midwives to re-balance the doctor:nurse ratio in public facilities, particularly at the district level.

Absenteeism of doctors is another concern for health facilities [10]. A study in Bangladesh by World Bank found a 40% absenteeism rate for doctors in sub-district-level hospitals and a 74% in union health centers [10]. However, no published data on absenteeism rates at the DHs and MCWCs in Bangladeshis available. Above all, an inequitable distribution of health personnel is present in Bangladesh with an oversupply of medical doctors in the urban areas and an undersupply in rural and hard-to-reach areas [6]. Many healthcare providers become frustrated when working in rural placements because of a lack of equipment and supplies along with means of healthy living [14]. If the equipment and infrastructure of the workplace were ensured, doctors might be more encouraged to work [15]. A study in Vietnam documented that in addition to salaries and allowances, working conditions, availability of equipment, communications and relationships with colleagues, appreciation by their managers, and communities as well as provision of training for personal development were important factors in attracting and retaining health workers [16, 17].

Other modalities to fill shortages of health care providers can be tried. For instance, India has focused on public-private partnerships to fill vacant posts by hiring health care workers from private sector. In the states of Karnataka and Arunachal Pradesh, the Government of India has authorized public facilities to contract with NGOs to provide services [18]. In another state of India, Haryana, decentralized recruitment of specialist doctors has been tried as of location-specific recruitment in underserved areas of West Bengal [18]. In Thailand, a rural recruitment system, training provided in rural health facilities, hometown placement, and performance-based agreements have been successfully used to increase retention of providers and improve the distribution of health workers [19]. A study in Tonga showed that a regular rotation from rural and remote to urban posts improved professional development, sharing of skills among providers, and job satisfaction by providing a way out for those who felt entrapped in a rural setting [19].

There are strategies in the national health policy of Bangladesh to ensure retention of doctors in their posted locations to ensure round-the-clock availability of services in rural and remote areas by increasing the accountability of the providers to the community [20]. The national health policy also includes regulations for quality of care and ethical medical practices in both public and private facilities that require monitoring and evaluation of services. Unfortunately, this policy is not implemented, and the health system governance is too weak to enforce the regulations that hold health workers accountable for their performance. Use of our tool may provide a relatively easy method of measuring staffing needs to increase accountability.

This study had some limitations. First, the measurement of shift coverage was divided into only two parts (more than 50% and less than 50% of the duty period) instead of allowing for calculations of coverage over smaller periods of time. Secondly, all types of providers were counted equally in the index; no priority of provider type was set, while provision of services may be dependent on the skill of available staff. Thirdly, our definition of at least one medical doctor per shift may not be sufficient to meet patient load. Fourthly, we considered the presence of the service providers in the facility in response to emergency call but did not take into account any delay in arrival of the doctor to attend the patient. Further, these indices only measure presence of staff, but do not assess competence/quality of care.

## Conclusion

To quantify the availability of health care providers at facilities is complex due to the nature of service and diversity of care providers. Our human resource availability index has the potential to be used as a tool to identify shortages at the facility level, to increase provider accountability and to advocate for policies to reduce human resources gaps for EmONC services. Improving the availability of these services is needed to reduce maternal mortality to achieve sustainable development goals. The proposed approach for staff availability indices is simple and quick to implement. To the best of our knowledge, this is the first study providing a method for measuring the availability of providers for EmONC services. Further studies are needed in a larger sample of different health facilities for adaptation of this tool, and to test how it can be used to enhance motivation of providers to ensure 24 h EmONC services.

## Abbreviations

ANA: Assistant Nursing Attendant
DH: District Hospital
EmONC: Emergency Obstetric and Newborn Care
FWV: Family Welfare Visitor
MCWC: Mother and Child Welfare Centre
MLSS: Member of lower subordinate staff
MNH: Maternal and Newborn Care
MT: Medical Technologist
Ob/gyn: Obstetrics/gynecology; WHO: World Health Organization
